# Mechanisms Underlying the Functional Cooperation Between PPARα and GRα to Attenuate Inflammatory Responses

**DOI:** 10.3389/fimmu.2019.01769

**Published:** 2019-08-09

**Authors:** Nadia Bougarne, Viacheslav Mylka, Dariusz Ratman, Ilse M. Beck, Jonathan Thommis, Lode De Cauwer, Jan Tavernier, Bart Staels, Claude Libert, Karolien De Bosscher

**Affiliations:** ^1^Translational Nuclear Receptor Research Lab, Ghent, Belgium; ^2^Department of Biomolecular Medicine, Ghent University, Ghent, Belgium; ^3^VIB Center for Medical Biotechnology, Ghent, Belgium; ^4^Receptor Research Laboratories, Cytokine Receptor Lab, Ghent, Belgium; ^5^Univ. Lille, Inserm, CHU Lille, Institut Pasteur de Lille, U1011 - EGID, Lille, France; ^6^Department of Biomedical Molecular Biology, Ghent University, Ghent, Belgium; ^7^VIB Center for Inflammation Research, Ghent, Belgium

**Keywords:** PPARα, GRα, crosstalk, molecular mechanism, inflammation, MSK1

## Abstract

Glucocorticoids (GCs) act via the glucocorticoid receptor (NR3C1, GRα) to combat overshooting responses to infectious stimuli, including lipopolysaccharide (LPS). As such, GCs inhibit the activity of downstream effector cytokines, such as tumor necrosis factor (TNF). PPARα (NR1C1) is a nuclear receptor described to function on the crossroad between lipid metabolism and control of inflammation. In the current work, we have investigated the molecular mechanism by which GCs and PPARα agonists cooperate to jointly inhibit NF-κB-driven expression in A549 cells. We discovered a nuclear mechanism that predominantly targets Mitogen- and Stress-activated protein Kinase-1 activation upon co-triggering GRα and PPARα. *In vitro* GST-pull down data further support that the anti-inflammatory mechanism may additionally involve a non-competitive physical interaction between the p65 subunit of NF-κB, GRα, and PPARα. Finally, to study metabolic effector target cells common to both receptors, we overlaid the effect of GRα and PPARα crosstalk in mouse primary hepatocytes under LPS-induced inflammatory conditions on a genome-wide level. RNA-seq results revealed lipid metabolism genes that were upregulated and inflammatory genes that were additively downregulated. Validation at the cytokine protein level finally supported a consistent additive anti-inflammatory response in hepatocytes.

## Introduction

Glucocorticoid hormones (GCs) are the mainstay of treatment for most inflammatory and autoimmune diseases (1, 2). GCs also regulate glucose and fat homeostasis, however a long-term therapeutic treatment with exogenous GCs causes hyperglycaemia, insulin resistance and disturbed fat profiles as clinically worrying drawbacks (3). A reduction in adverse effects related to glucose and fat regulation would be highly desirable in clinical GC applications.

Therapeutic activities of GCs are mediated by the glucocorticoid receptor (NR3C1) (4), belonging to the superfamily of ligand-inducible transcription factors (4). Unliganded GR predominantly resides in the cytosol in an inactive state associated with heat shock proteins (HSPs) and immunophilins (4, 5). Upon GC binding, GR translocates to the nucleus and binds to GR binding sequences (GBSs), widely dispersed throughout the genome (6). These may include enhancers, hot spots, as well as GC-response elements (GREs) within the promoter regions of target genes, hereby regulating their transcriptional activity (7–10). Additionally, transcriptional regulation mediated by the GR also encompasses inhibitory effects on the activity of pro-inflammatory transcription factors driving the onset of inflammation, such as nuclear factor-κB (NF-κB), resulting in pro-inflammatory gene suppression (11–13). Throughout the years, many different mechanisms have been proposed explaining how GR inhibits pro-inflammatory gene expression, including direct mechanisms as well as feedback loop mechanisms by GC-induced anti-inflammatory proteins (14, 15). Suggestive of conserved mechanisms among nuclear receptors, the fibrate ligand-activated transcription factor peroxisome proliferator-activated receptor α (PPARα), a member of the nuclear hormone receptor superfamily, may also exert anti-inflammatory actions by down-regulating the activity of NF-κB and other pro-inflammatory transcription factors via multiple mechanisms, with some reminiscent of the ones GR is deploying (16, 17).

In addition, both GR and PPARα exhibit overlapping and complementary roles in liver with regard to carbohydrate and fat metabolism (13, 18) and co-ordinately control key genes involved in the maintenance of blood glucose levels, cooperatively support fatty acid β-oxidation during fasting, and stimulate immune suppression (19–21).

We previously reported that GRα and PPARα, when co-activated, physically interact *in vitro* and *in cellulo*, in the nucleus (22), paving the way for an extra level of gene regulatory mechanisms apart from triggering their own cognate gene programs. PPARα activation further enhanced GR-triggered suppression of TNF-induced NF-κB-driven gene expression and pro-inflammatory cytokine production in fibroblast (L929sA) cells (22). PPARα activation also suppressed GR-induced upregulation of *G6PC* (22), one of the metabolic genes responsible for adverse effects related to glucose metabolism upon chronic GC therapy. Mice subjected to a 7-week high fat diet and that received a daily administration of the synthetic GC Dexamethasone (DEX) for another 7 days instead of solvent, demonstrated a worsened glucose intolerance which coincided with enhanced hyperinsulinemia. Oppositely, high fat diet fat mice receiving the PPARα agonist fenofibrate (FENO) for 7 days supported clear glucose tolerance. Remarkably, the latter phenotype was also observed when combining DEX with FENO, indicating crosstalk and a potential advantage at the glucose metabolism level when combining two nuclear receptor ligands for which anti-inflammatory actions had been demonstrated (22). Collectively, these results justify further mechanistic exploration of a combination of GCs with PPARα agonists in a context of inflammation, starting with simple cell models to understand first the cell-autonomous crosstalk modes in more detail.

Mitogen- and Stress-activated protein Kinase-1 (MSK1) is a kinase that acts, among others, in the TNF-signaling pathway. It promotes inflammatory gene transcription by phosphorylating NF-κB, which facilitates association of p65 with cofactors, and by phosphorylating histone H3 (23–25). We previously reported that GCs counteract MSK1 recruitment at inflammatory gene promoters and partially drive MSK1 to the cytoplasm, as a contributory mechanism to inhibit NF-κB transactivation (23).

Crosstalk between GCs and MAPK signaling pathways was considered before as a valid mechanism to effectively inhibit NF-κB-driven inflammatory gene promoters (26). PPARα agonists have also been shown to modulate MAPK activities, indirectly suppressing inflammatory responses (27, 28). As we previously observed no significant inhibitory effect of GCs on p38 and ERK MAPK activation in L929sA mouse fibroblasts (29) and A549 human epithelial cells (23), we explored whether in A549 human epithelial cells combined treatment of GCs and PPARα agonists might target the more downstream kinase MSK1 and thus might contribute to the additive transrepression of NF-κB-driven inflammatory genes observed when triggering both receptors.

In the present research we overlaid a mechanistic study of the effect of GR and PPARα crosstalk under TNF-induced inflammatory conditions in A549 human epithelial cells as a first cellular model system for inflammatory responses, with a genome-wide impact of combined ligand treatment in metabolic effector cells using LPS-induced primary hepatocytes as a second, complementing, model system. RNA-seq results in primary hepatocytes revealed inflammatory genes that were synergistically downregulated and lipid metabolism genes that were additively upregulated following the activation of both nuclear receptors. In addition, our data reveal that, upon co-triggering of GRα and PPARα, a nuclear anti-inflammatory mechanism may follow from a hampering at the level of TNF-activated kinase MSK1 activation in a lung epithelial cell line. Taken together, our findings unveil novel molecular aspects of the PPARα-GR-mediated NF-κB-targeting anti-inflammatory mechanism.

## Materials and Methods

### Cytokines, Plasmids, and Reagents

Dexamethasone (D4902) (DEX) and GW7647 (G6793) (GW) were obtained from Sigma–Aldrich (St. Louis, MO, USA). Anti-GR, anti-PPARα, anti-RNA pol II and anti-p65 antibodies were obtained from Santa Cruz. Phospho-specific rabbit antibodies to p38 (Thr-180/Tyr-182), p42/44 ERK (Thr202/Tyr204), MSK1 (Thr581) and IKKα/β (Ser180/S181) were used to detect the respective phosphorylated forms and purchased from Cell Signaling. Anti-p38, anti-ERK, anti-MSK1, and anti-IκBα antibodies were purchased from Cell Signaling. Anti-tubulin and anti-actin were used as loading control and obtained from Santa Cruz. Anti-phospho-65 was obtained from Santa Cruz. Recombinant murine TNFα was produced and purified as described (30). TNFα was used at a final concentration of 2,000 IU/ml. p(IL6κB)_3_50hu.IL6P-luc+ (hereafter renamed NF-κB-Luc), PPARα, GR, and 5HT7 control plasmids were described previously (21, 31–33). LPS was purchased from Invivogen.

### Cell Culture

A549 cells were grown in DMEM plus 10% fetal calf serum, 100 U/ml penicillin and 0.1 mg/ml streptomycin. Cells were maintained in a 5% CO_2_-humidified atmosphere at 37°C.

### Transfection and Reporter Assays

A549 cells were transiently transfected using Lipofectamine and PLUS reagents, as described by the manufacturer (Invitrogen, Life Technologies). In short, cells within each well of a 24-well plate were transfected using 400 ng DNA, 1.2 μl lipofectamine and 0.8 μl PLUS reagent. After 5 h incubation with the transfection reagent, the medium was refreshed with standard culture medium (see above). After transfection, cells were left to rest for another 24 h before inductions. Cells were induced as indicated in the figure legends, after which luciferase assays were carried out according to instructions of the manufacturer (Promega). Luciferase measurements were performed at least in triplicate and normalized by measurement of β-galactosidase levels using the Galacto-Light kit (Tropix). Results presented are from 3 independent biological replicates.

### Western Analysis

Total cell lysates were prepared using 1 × SDS sample buffer (50 mM Tris pH 6.8; 2% SDS; 10% glycerol; bromophenol blue and 100 mM DTT, freshly added). Samples were incubated at 95°C for 5 min and separated on a SDS-PAGE gel and subsequently blotted onto a Nitrocellulose membrane (Whatman, Dassel, Germany). Immunoblotting was performed according to the standard protocol of Santa Cruz (Santa Cruz, CA, USA). Imaging of antibody-tagged protein signal was obtained via Western Lightning (PerkinElmer, Waltham, MA, USA). To quantify bands obtained via Western analysis, we applied band densitometric analysis via ImageJ software (http://rsb.info.nih.gov/ij/). The area under curve (AUC) of the specific signal of the protein of interest as indicated in the figure legend was corrected for the AUC of the loading control, indicated in the figure legend. Results representative of 2 independent biological repeats are shown.

### Immunofluorescence

Indirect immunofluorescence was performed as previously described (34). In short, A549 cells, seeded on coverslips and serum-deprived for 48 h, were induced as indicated in the figure legends. After fixation, endogenous p65 and MSK1 were visualized using the corresponding rabbit antibodies followed by Alexa Fluor 488 or Alexa Fluor 568 anti-rabbit IgG (Molecular Probes, Invitrogen). Endogenous PPARα was visualized using the corresponding goat antibody followed by Alexa Fluor 488 anti-goat IgG (Molecular Probes, Invitrogen). Endogenous GRα was visualized using the corresponding mouse antibody followed by Alexa Fluor 568 anti-mouse IgG (Molecular Probes, Invitrogen). Cell nuclei were stained using DAPI DNA staining (300 nM, Invitrogen).

### *In vitro* Protein-Protein Interaction Assay (GST Pull-Down)

GST-fusion proteins with PPARα and 5HT7 were expressed in BL21 bacterial cells and purified with glutathione-agarose beads. GRα and p65 proteins were transcribed and translated *in vitro* using the TNT T7-coupled reticulocyte lysate system (Promega) according to the manufacturer's instructions. GST pull-down was carried out by incubating the equivalent of 2 μg of GST-PPARα beads with 10 μl of *in vitro* translated [^35^S]-methionine labeled GRα with increasing amounts of non-labeled GRα, or by incubating the equivalent of 2 μg of GST-PPARα beads with 10 μl of [^35^S]-methionine labeled p65 with increasing amounts of [^35^S]-methionine labeled GRα or finally, by incubating the equivalent of 2 μg of GST-PPARα beads with 10 μl of [^35^S]-methionine labeled GRα with increasing amounts of [^35^S]-methionine labeled p65. All of these interaction studies were performed in a total volume of 200 μl of incubation buffer [20 mM Tris-HCl (pH 8), 300 mM NaCl, 6 mM MgCl_2_, 8% glycerol, 0.05% Nonidet P-40, 0.1% dithiothreitol]. The mixture was gently rotated for 2 h at 4°C. After centrifugation, the beads were washed five times with incubation buffer supplemented with NaCl up to a final concentration of 500 mM, next resuspended in 25 μl of 1x Laemmli buffer, boiled for 3 min, and centrifuged. After GST-mediated purification and extensive washes, proteins were separated on polyacrylamide gels and visualized by autoradiography. GST-5HT7 was used as a negative control.

### Primary Hepatocyte Isolation

Primary hepatocytes were isolated from 10 to 12 week-old male C57BL/6 mice by collagenase perfusion (35). The procedure was modified by excluding insulin and DEX supplementation in the William's medium (Sigma, W1878), but keeping 0.1% free-fatty acids and 1% glutamine. After isolation cells were seeded on collagen-coated 6-well plates at a density of 0.75 × 10^6^ cells. After 2 h of attachment medium was refreshed and ligands were introduced, as indicated in the figure legends.

### qPCR and ChIP-qPCR

RNA was isolated with the RNeasy purification kit (Qiagen) according to the user manual. cDNA was synthesized with a PrimeScript kit (Takara). qPCR was performed using Light Cycler 480 SYBR Green I Master Mix (Roche). The primer list is provided in Table S1. qPCR data were normalized and quantified relative to the 2 most stable reference genes with qbase+ (36). ChIP assays were performed as previously described (37). The relative amount of the precipitated target sequence was determined via normalization to the “input”, i.e., the purified total gDNA levels. The primers for IL8, encompassing −121/+61, have been described earlier (38).

### RNA-Seq Analysis

RNA-seq was done in three biological replicates. Each replicate was obtained by pooling cells from 3 to 4 mice and then performing induction in three technical replicates. RNA was isolated with the RNeasy purification kit (Qiagen) according to the user manual. Library preparation and sequencing was prepared by the VIB Nucleomics Core facility. 75 bp long sequenced reads were generated with Illumina NextSeq 500 and were mapped to the mm10 genome using tophat (version 2.0.11). Gene counts were calculated with htseq-count (0.6.1) using “intersection-strict” mode. Gene level differential expression analysis was performed with the aid of the R package “DESeq2” by applying the following contrasts (*p* adjusted <0.05): LPS vs. DEX+LPS, LPS vs. GW+LPS, LPS vs. DEX/GW+LPS, DEX+LPS vs. DEX/GW+LPS and GW+LPS vs. DEX/GW+LPS. Differentially expressed genes were combined into a single list and re-ordered using a K-mean clustering (6 clusters). Gene ontology analysis of gene clusters 2, 3, and 5 was performed using “goseq” R package.

## Elisa

CCL2 and IL6 ELISA was performed on media from primary hepatocytes after 19 h induction with compounds DEX and/or GW in combination with 100 ng/ml LPS by using the ELISA MAX Standard (BioLegend, 432702, 430502), in according with the manual.

### Statistical Analysis

Statistical analysis was performed using the GraphPad Prism software (version 7.02 or 8). Significant differences between groups were evaluated using two-way (2 factors) ANOVA with Dunnett's test for multiple comparison, which was found to be appropriate as groups displayed a normal distribution. Normality was tested with the D'Agostino-Pearson normality test. When variances across groups were not equal, logarithmic transformation was applied prior to statistical analysis. Values are expressed as mean + SEM, and error bars were derived from biological replicates rather than technical replicates. *p* < 0.05 was considered statistically significant.

## Results

### GCs and PPARα Agonists Inhibit Pro-inflammatory Gene Expression in a Concentration-Responsive Manner

**Figure 1:**
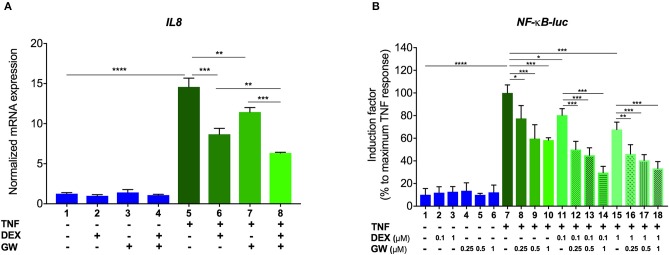
GCs and PPARα agonists inhibit pro-inflammatory gene expression in A549 cells. **(A)** A549 cells were pre-incubated with solvent, DEX (1 μM), GW (0.5 μM) or various combinations thereof, for 1 h, before TNF (2000 IU/ml) was added, where indicated, for a total induction time of 6 h. mRNA was isolated, reverse transcribed, and subjected to QPCR using primers to detect IL8. qPCR measurements were performed in triplicates. qPCR results, normalized to expression of household genes, are shown ± SD. **(B)** A549 cells were transiently transfected with NF-κB-Luc using Lipofectamine/Plus reagents, as described (Invitrogen, Carlsbad, CA, USA). 24 h after transfection, cells were incubated with solvent, DEX (0.1 or 1 μM), GW (0.25, 0.5, or 1 μM) or various combinations thereof, for 1 h, before TNF (2000 IU/ml) was added, where indicated, for a total induction time of 6 h. Cell lysates were assayed for luc activities and normalized with β-gal activities. Promoter activities are expressed as relative induction factor calculated as percentage of maximal TNF response. Results in **(A,B)** are from three independent biological replicates (*n* = 3) with measurements in triplicate. Statistical analysis was done using ANOVA with Tukey's multiple comparison post-test (^*^p < 0.05, ^**^p < 0.01, ^***^p < 0.001, ^****^p < 0.0001).

We first verified, using A549 lung epithelial cells, that the single PPARα agonist GW7647 (hereafter GW) and the single synthetic GR agonist dexamethasone (DEX) are both able to inhibit TNF-induced gene expression (Figure 1A, lanes 6 and 7 compared to lane 5). We go on to show that an additive anti-inflammatory effect can be observed for a complex NF-κB-driven promoter in its endogenous promoter context, i.e., TNF-induced IL-8 mRNA expression (Figure 1A, lane 8 compared to lanes 6 and 7). Results from A549 cells transiently transfected with a recombinant NF-κB-driven promoter construct as a direct transcriptional read-out (Figure 1B) confirm TNF-induced NF-κB as a relevant nuclear receptor target and show anti-inflammatory effects by single DEX and GW, in a concentration-responsive manner (Figure 1B, lanes 8 to 10 and lanes 11 and 15 compared to lane 7). Combined DEX/GW treatment results in an additive repression of TNF-induced recombinant NF-κB promoter activity when compared to compound alone (Figure 1B, lanes 12 to 14 compared to lane 11 and lanes 16–18, compared to lane 15) even when using saturating amounts of DEX. Taken together, these data support our previous findings in L929sA where the additive anti-inflammatory effect of DEX and GW also converged on NF-κB (22). Collectively, these results raise the question whether combined ligand treatment may act differently on components of the upstream cascade leading toward NF-κB or may differently impinge on NF-κB binding or activity.

### Co-activation of GRα and PPARα Does Not Affect the Upstream TNF-Induced IKK Activation Pathway or the Nuclear Accumulation of Activated p65

**Figure 2:**
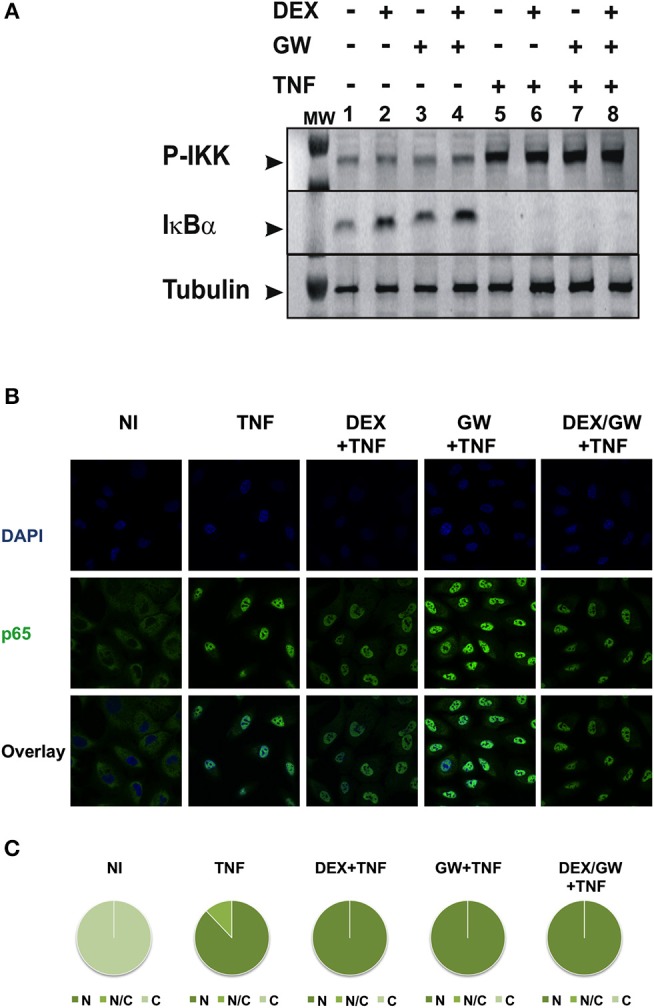
Co-activation of GRα and PPARα does not affect pathways influencing the nuclear accumulation of activated p65. **(A)** A549 cells, starved for 48 h in DMEM devoid of serum, were pretreated with solvent, DEX (1 μM), GW (0.5 μM) or various combinations for 1 h, before TNF (2000 IU/ml) was added, where indicated, for 30 min. Cell lysates were subjected to western blotting using anti-phospho-IKK or anti-IκBα antibodies, and using anti-tubulin as a loading control, as indicated. A representative blot of *n* = 2 is shown. **(B)** A549 cells were treated with DEX (1 μM) and/or GW (0.5 μM) and/or TNF (2000 IU/ml). Indirect immunofluorescence was performed using an anti-p65 antibody. Endogenous p65 was visualized (green), DAPI staining indicates the nuclei of the cells (blue) and “Overlay” shows a merged image with both stainings combined. Representative images of *n* = 2 are shown. **(C)** Per induction, minimally three random fields of minimally 5 cells/field were scored. Scored cells are categorized into three groups according to the subcellular distribution of p65, i.e., C, mainly cytoplasmic; N, mainly nuclear; N/C, equally distributed (nuclear/cytoplasmic) with % distribution presented as pie charts.

To first test whether the TNF-induced kinase cascade upstream of the activity of p65 can be a target of a GRα and PPARα-mediated inhibition, we evaluated levels of activated IKK and the inhibitory protein of NF-κB. IκBα is known to be degraded following activation of IKK and subsequent phosphorylation upon an inflammatory stimulus, e.g., TNFα. This was confirmed in Figure 2A (for quantification please see Figure S1). No significant effect of DEX, GW or the combination hereof was apparent on TNF-activated IKK (Figure 2A). In line with these results, DEX and GW also did not affect the TNFα-induced nuclear translocation of the p65 subunit of NF-κB as shown by indirect immunofluorescence analysis (Figure 2B). Based on these results, the cooperative anti-inflammatory activity of GCs and PPARα agonists most likely operates within the cellular nucleus.

### Co-activation of GRα and PPARα Does Not Affect MAPK Activation but Efficiently Lowers Levels of Phospho-MSK-1 in A549

**Figure 3:**
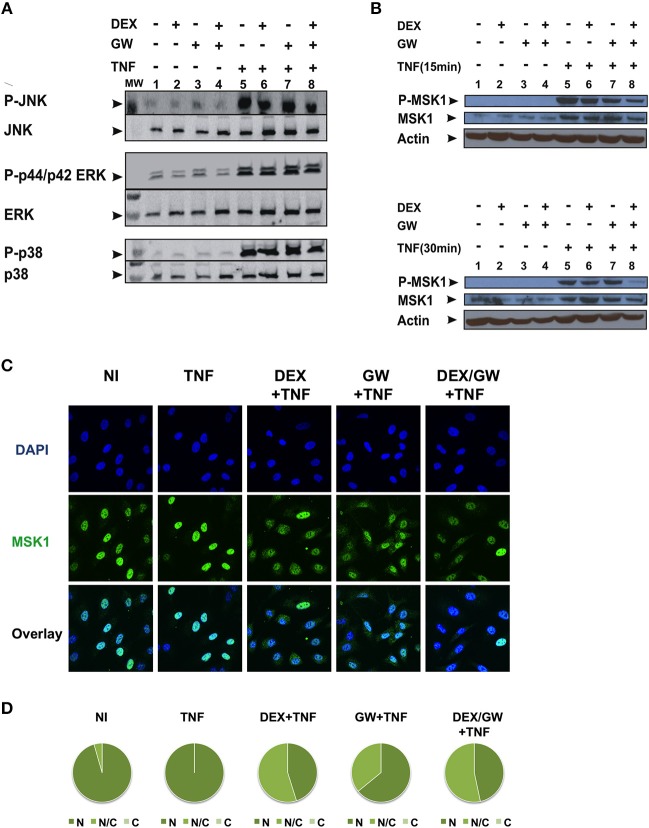
Co-activation of GRα and PPARα efficiently lowers levels of phospho-MSK-1 in A549. **(A)** A549 cells, starved for 48 h in DMEM devoid of serum, were pretreated with solvent, DEX (1 μM), GW (0.5 μM) or various combinations for 1 h, before TNF (2000 IU/ml) was added, where indicated, for 30 min. Cell lysates were subjected to western blotting with anti-phospho-MAPK and the corresponding non-phospho antibodies; for this re-probed blot the same overall loading control applies as shown in Figure 2A. A representative blot of *n* = 2 is shown. **(B)** A549 cells, starved for 48 h in DMEM devoid of serum, were pretreated with solvent, DEX (1 μM), GW (0.5 μM) or various combinations for 1 h, before TNF (2000 IU/ml) was added, where indicated, for 15 min and 30 min. Cell lysates were subjected to western blotting with anti-phospho-MSK1, anti-MSK1 and anti-actin as a loading control, as indicated. A representative blot of *n* = 2 is shown. **(C)** A549 cells were treated with DEX (1 μM) and/or GW (0.5 μM) and/or TNF (2000 IU/ml) for 30 min. Indirect immunofluorescence was performed using an anti-MSK1 antibody. Endogenous MSK1 was visualized (green), DAPI staining indicates the nuclei of the cells (blue) and Overlay indicates an image of both stainings combined. Representative images of *n* = 2 are shown. **(D)** Per induction, minimally three random fields of minimally 5 cells/field were scored. Scored cells are categorized into three groups according to the subcellular distribution of MSK1, i.e., C, mainly cytoplasmic; N, mainly nuclear; N/C, equally distributed (nuclear/cytoplasmic) with % distribution presented as pie charts.

As we observed no significant inhibitory effect of combined DEX/GW treatment on the above-mentioned kinases in Figure 2, we further explored whether combined treatment of GCs and PPARα agonist might target TNF-induced phospho-ERK, phospho-JNK and phospho-p38 or the downstream nuclear kinase MSK1 (Figure 3). As shown in Figure 3A, none of the TNF-activated MAPK is differentially affected comparing GC/PPARα co-treatment with single treatments (for quantification please see Figure S2A). However, compared to each compound alone, co-treatment with the PPARα agonist GW and DEX clearly reduces the TNF-induced MSK1 phosphorylation, apparent at 15 min (Figure 3B, upper panel) and at 30 min (Figure 3B, lower panel) (for quantification please see Figure S2B). In line with our previous results (23), DEX is able to partially extrude TNF-induced MSK1 from the nucleus (Figure 3C). Both GW alone as well as the combination DEX/GW yields a similar result when combined with TNF, as compared to TNF alone (Figure 3C). From the cell counts it is clear that combined DEX/GW with TNF recapitulates the same phenotype as observed for DEX/TNF (Figure 3D). Still, in all combinations a predominant nuclear MSK1 signal remains. Taken together, these results suggest that the combined inhibitory effect of GCs and PPARα agonists on phosphorylated MSK1 may contribute to the additive transrepression of NF-κB-driven inflammatory genes triggered by activated GR and PPARα.

### Ligand-Activated GRα and PPARα Are Both Localized in the Nucleus in TNF-Stimulated A549 Cells

**Figure 4:**
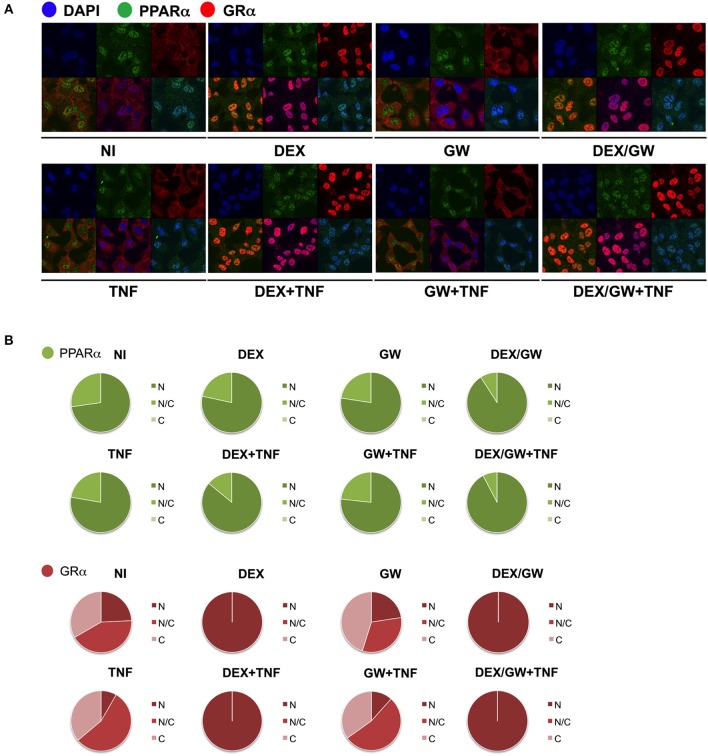
Ligand-activated GRα and PPARα are both localized in the nucleus in TNF-stimulated cells. **(A)** A549 cells, starved for 48h in DMEM devoid of serum, were pretreated with solvent, DEX (1 μM), GW (0.5 μM) or various combinations for 1h, before TNF (2000 IU/ml) was added, where indicated, for 30 min. Localization of PPARα (green) and GRα (red) was assessed by confocal analysis. DAPI staining indicates the nuclei of the cells (blue). Immunofluorescence of representative cell fields are shown (*n* = 1). **(B)** Per induction, minimally three random fields of minimally 5 cells/field were scored. Scored cells are categorized into three groups according to the subcellular distribution of PPARα (green) and GRα (red), i.e., C, mainly cytoplasmic; N, mainly nuclear; N/C, equally distributed (nuclear/cytoplasmic).

We next wondered whether the activated nuclear receptors would remain nuclear in absence and presence of TNF. Endogenous co-immunolocalization analyses show that under conditions in which p65 is activated upon TNF (Figures S3, S4) and under conditions when both GRα and PPARα are activated, the latter proteins effectively reside predominantly in the nuclear compartment (Figure 4).

### Combined DEX and PPARα Agonist Treatment Maintains Chromatin Recruitment of TNF-Activated p65

**Figure 5:**
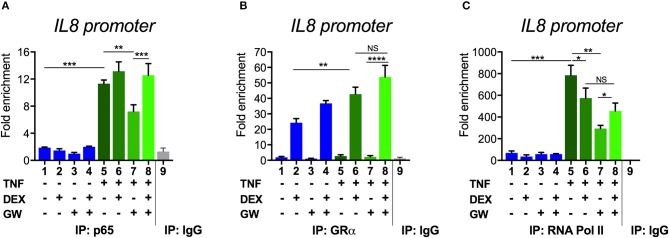
Combined DEX and PPARα agonist treatment maintains chromatin recruitment of TNF-activated p65. Following serum starvation for 48 h, A549 cells were pre-incubated with solvent, DEX (1 μM), GW (0.5 μM) or various combinations for 1 h, before TNF (2000 IU/ml) was added, where indicated, for 30 min. Cross-linked and sonicated cell lysates were subjected to ChIP analysis against p65 **(A)**, GR **(B)** or RNA pol II **(C)**. qPCR was used to assay recruitment at the IL8 gene promoter. The quantity of p65, GR or RNA pol II detected at the IL8 promoter is shown with a correction of the SYBR green qPCR signal for input control. Lanes 1–8 contain data derived from DNA pulled with specific antibody-prepared ChIPs, as indicated in the graph; lane 9 includes the IgG control. The reaction was performed in triplicate. Results are compiled from three independent biological replicates (*n* = 3). Statistical analysis was done using ANOVA with Tukey's multiple comparison post-test. (^*^p < 0.05, ^**^p < 0.01, ^***^p < 0.001, ^****^p < 0.0001).

To next study the impact of single vs. combined ligand treatment on the subsequent binding behavior of NF-κB we analyzed the IL8 promoter nearby the promoter proximal NF-κB binding site, using chromatin immunoprecipitation (ChIP) analysis. The results in Figure 5A show that the PPARα agonist GW alone reduces the TNF-induced p65 recruitment at this inflammatory promoter, however, single DEX or combined DEX/GW treatment clearly does not affect TNF-induced promoter occupation of p65. When analyzing concomitant GR occupancy under the same conditions, DEX treatment consistently increases GR recruitment at the IL8 promoter (Figure 5B). When combined with TNF, DEX supports even more GR recruitment (Figure 5B, compare lanes 2 and 6). Of note, additional GW treatment does not further affect GR recruitment (Figure 5B, lane 8). In concordance with the results on gene repression (Figure 1), we detect lower IL8 promoter occupancy of RNA polymerase II (RNA pol II) when combining DEX, GW or DEX/GW as compared to TNF alone (Figure 5C). The combination of DEX/GW with TNF did however not result in a lower IL8 promoter occupancy of RNA pol II as compared to DEX/TNF, or GW/TNF alone. Lower levels of RNA pol II recruitment upon GW/TNF (Figure 5C) nicely correlate with a lower level of p65 recruitment upon GW/TNF (Figure 5A), yet again the effect of DEX, and additional presence of GR (Figure 5B) is dominant. Taken together, these results show that even though MSK1 activation is reduced (Figure 3B), still, p65 is not dissociated from the IL8 promoter under conditions of a maximal pro-inflammatory gene inhibition by DEX and PPARα agonists.

### PPARα and GRα Interact With NF-κB p65 in a Non-competitive Manner *in vitro*

The underlying mechanism as suggested by the transcriptional data (Figure 1) and the ChIP results (Figure 5) may involve either tethering events or independent DNA binding events. Direct interactions between single GR or single PPARα with the p65 subunit of NF-κB were previously reported to contribute to the inhibition of NF-κB-dependent pro-inflammatory gene expression and were described to involve (a) the DNA binding domain of either GRα or PPARα and (b) the Rel Homology Domain (RHD) of p65 (33, 39, 40). To obtain further insight into the molecular basis of the additive anti-inflammatory effect observed upon combining GR and PPARα agonists, we tested whether GRα and PPARα are able to bind p65 simultaneously or instead in a competitive and mutually exclusive manner. Since both receptors have been described to interact with largely similar domains within p65 (AA 22-248 and 12-378 for GRα and PPARα, respectively (33, 39, 40), the possibility of a competitive and independent binding was considered.

**Figure 6:**
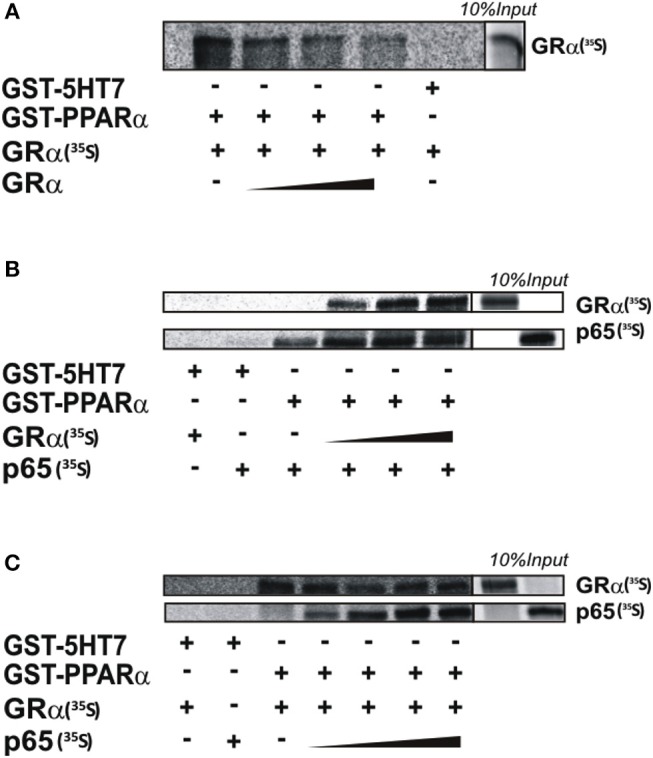
PPARα and GRα interact with NF-κB p65, in a non-competitive manner *in vitro*. GST-fusion proteins PPARα and 5HT7 were expressed in BL21 bacterial cells and purified with glutathione-agarose beads. [S35]-methionine labeled GRα and or p65 products were generated with TNT reaction, using rabbit reticulocyte lysates. **(A)** [^35^S]-methionine labeled GRα was incubated with Glutathione-Sepharose 4B beads loaded with GST-PPARα or GST-5HT7 as control with increasing amount of non-labeled GRα. **(B)** [^35^S]-methionine labeled p65 was incubated with Glutathione-Sepharose 4B beads loaded with GST-PPARα or GST-5HT7 as control with increasing amount of [^35^S]-methionine labeled GRα. **(C)** [^35^S]-methionine labeled GRα was incubated with Glutathione-Sepharose 4B beads loaded with GST-PPARα or GST-5HT7 as control with increasing amount of [^35^S]-methionine labeled p65. Representative images of *n* = 2 are shown.

GST-pull down experiments show that binding between GST-PPARα and *in vitro* produced GRα (^35^S) can be outcompeted by cold GRα (Figure 6A, quantification see Figure S5A), illustrating the feasibility to detect competitive binding in a GST-pull down assay and supporting our previous findings, via co-IP, that PPARα and GRα indeed physically interact (22). The interaction between GST-PPARα and *in vitro* produced p65 (^35^S) is however not affected by increasing amounts of GRα (^35^S) (Figure 6B, quantification see Figure S5B). Similarly, adding increasing amounts of p65 (^35^S) also does not affect the binding between GST-PPARα and *in vitro* produced GRα (^35^S) (Figure 6C, quantification see Figure S5C). Altogether, our GST-pull down experiments support that GR and PPARα may interact with the RHD of p65 in a non-competitive manner, supporting the hypothesis of complex formation between all three transcription factors.

The *in vitro* experiments cannot take into account the possibility that the single ligand treatments and/or co-treatments may additionally affect receptor protein expressions in a cellular environment. To address this extra parameter, A549 cells were pretreated with solvent, DEX (1 mM), GW (0.5 μM) or various combinations for 1 h, before TNF (2000 IU/ml) was added for a total induction time of 6 h (to match the time points in Figure 1). Interestingly, the results from Figure S6 show that in inflamed cells (last 4 lanes, with TNF added) the combined ligand treatment DEX/GW is capable of lowering not only protein levels of the pro-inflammatory protein p65, but concomitantly also of both receptor levels. Strikingly, GW/DEX alone largely recapitulated the effect observed of both ligands in presence of TNF. Similar data were found for a shorter time point (1.5 h) (Figure S7), albeit not as outspoken. These findings nevertheless support the validity of the findings presented in Figure S6.

### GR and PPARα Co-regulate Lipid Metabolism and Inflammatory Gene Expression in Opposite Manners in Inflamed Murine Hepatocytes

**Figure 7:**
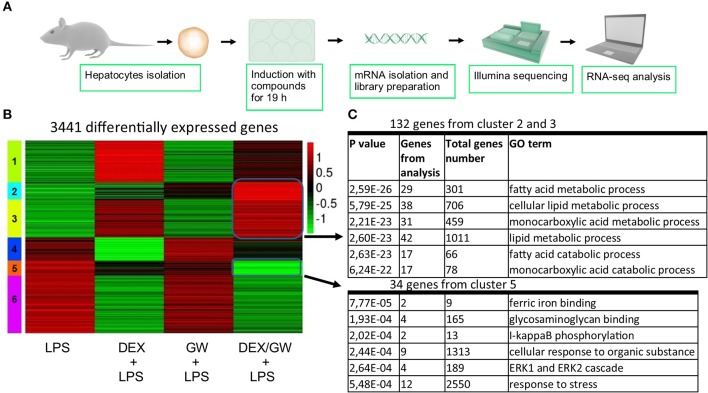
Co-activation of GRα and PPARα enhances lipid metabolism gene subsets and lowers stress response gene subsets in LPS-induced primary hepatocytes. **(A)** Schematic overview of the RNA-seq experiment (*n* = 3). **(B)** Heatmap using K-mean clustering of 3,441 differentially expressed genes from contrasts (p adjusted < 0.05): LPS (100 ng/ml) vs. DEX+LPS, LPS vs. GW+LPS, LPS vs. DEX/GW+LPS, DEX+LPS vs. DEX/GW+LPS and GW+LPS vs. DEX/GW+LPS. Color scale represents gene counts. **(C)** Gene ontology analysis of differentially upregulated (clusters 2 and 3) and downregulated (cluster 5) genes by DEX/GW vs. LPS, DEX and GW.

When looking at the broader picture of possible target cells, GCs and PPARα will not only regulate genes in immune or structural cell types coping with an inflammatory insult (e.g., synovial fibroblasts, macrophages, T-cells, or lung epithelial cells as studied here), but will also trigger gene programs in metabolic tissues, such as hepatocytes. Activated GR and PPARα have been described before to additively upregulate a vast subset of key genes of the lipid metabolism pathway in naïve murine primary hepatocytes (21). Combined ligand treatment was shown to exhibit anti-inflammatory capacities in lung epithelial cells as typical effector cells contributing to an inflammatory response (Figure 1), but it remained uncertain whether primary hepatocytes would behave in a similar manner, given a dominant role of GR/PPARα in glucose and fat metabolism in this cell type. To address this question, we performed RNA-seq following DEX and GW co-treatment for 19 h in presence of LPS to additionally mimic an inflamed state (Figure 7A). K-means clustering following the differential expression analysis revealed 992 genes (Figure 7B, cluster 2 and 3) upregulated by the combination of DEX/GW with LPS treatment compared to LPS alone. Among those, 132 genes were significantly more upregulated when compared to each compound alone (DEX + LPS or GW + LPS). Gene ontology analysis of these 132 genes attributed them to the lipid metabolism pathway (Figure 7C). This was consistent with previous results obtained in a basal state (21). LPS treatment did not influence DEX/GW-co-regulated gene expression in primary hepatocytes of one of the key co-controlled genes, *Angptl4*; a result that was independently validated by qPCR (Figure S8). We detected also 279 genes downregulated by DEX/GW + LPS treatment compared to LPS (Figure 7B, cluster 5). Only 34 of those were significantly more repressed upon comparing with either DEX + LPS or GW + LPS treatment alone. Some of these genes are inflammatory markers such as *Icam1, Ikbke, Nfkb2, Mapk3, Tlr2*.

### GR and PPARα Cooperate to Downregulate Inflammatory Genes and Proteins in Inflamed Murine Hepatocytes

**Figure 8:**
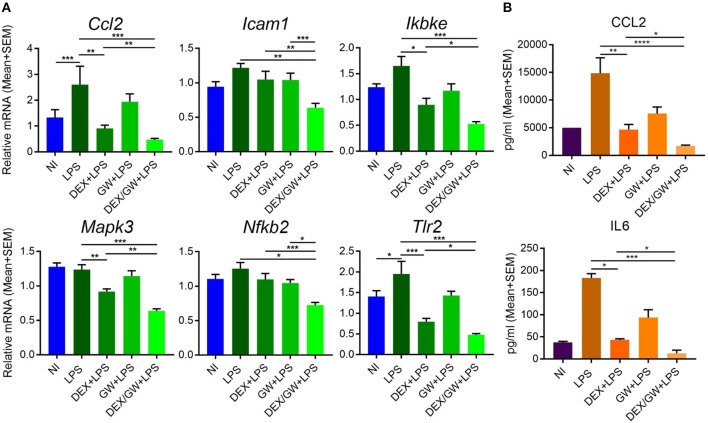
Co-activation of GRα and PPARα additively lowers inflammatory gene and protein expression in LPS-induced primary hepatocytes. Results are shown for mRNA **(A)** and protein **(B)** levels. **(A)** Following the treatment of primary hepatocytes as described in the legend of Figure 6, mRNA was isolated followed by qPCR analysis. Gene expression levels were normalized to Ppia/cyclophilin and Gapdh reference gene expression using qbase+ (*n* = 4–5). **(B)** CCL2 and IL6 ELISA from the media of primary hepatocytes after 19 h treatment with DEX (1 μM) and GW (0.5 μM) in combination with 100 ng/ml LPS (*n* = 3). Statistical analysis was done using 1-way ANOVA and Dunnett's test (^*^p < 0.05, ^**^p < 0.01, ^***^p < 0.001, ^****^p < 0.0001). NI, non-induced.

The results were next validated using qPCR in independently isolated murine primary hepatocytes (Figure 8A). We also determined mRNA levels of the classic inflammatory marker *Ccl2*. Similar to mRNA results, the protein levels of CCL2 were suppressed by combined DEX/GW treatment in presence of the inflammatory stimulus when compared to each compound alone (Figure 8B). Although the overall expression levels of IL6 in LPS-induced hepatocytes were almost two orders of magnitude lower than of CCL2 levels, we still observed a similar regulation (Figure 8B). Taken together, in analogy with the TNF-induced lung epithelial cell model, simultaneous GR and PPARα activation also supports additive anti-inflammatory effects in the LPS-inflamed primary hepatocyte model.

## Discussion

**Figure 9:**
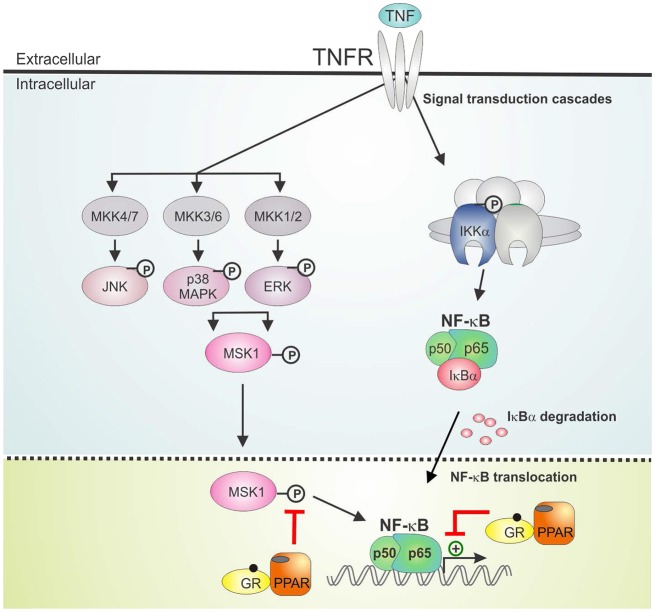
GRα/PPARα controlled points of interference with the TNF signaling pathway. Graphical abstract demonstrating activated GRα/PPARα efficiently inhibits TNF-driven gene expression in A549 cells primarily by interfering with the phosphorylation status of MSK1. Signaling components of the NF-κB pathway that have been studied in the manuscript are shown in non-gray colors. GR, Glucocorticoid Receptor α; IKK, IκB kinase, MAPK, Mitogen-activated protein kinase; MKK, MAP kinase kinase; PPAR, peroxisome proliferator activated receptor α; TNF, tumor necrosis factor; TNFR, TNF receptor.

The activation of PPARα was shown before to suppress the induction of liver gluconeogenic *G6PC* and *PEPCK* genes that were activated by GR in mice subject to a high fat diet (22). As such, combined PPARα and GRα agonist treatment might hold a promise of therapeutic benefit when able to cooperatively enhance anti-inflammatory effects, while circumventing (at least) the side effect of GC-induced glucose intolerance. In the current research we studied the GRα-PPARα crosstalk paradigm and its putative role in the transcriptional regulation of inflammatory genes comparing two cell types in which both GRα and PPARα are well-expressed and functional, i.e., hepatocytes and lung epithelial cells. We demonstrated that simultaneous GRα-PPARα activation additively suppresses inflammation both in LPS-treated murine primary hepatocytes and TNF-induced human lung epithelial cells. In the latter cell type, we went on to show via Western analysis using phospho-specific antibodies, that GR-PPARα crosstalk may block inflammatory cytokine gene expression in the nucleus by mitigating the activity of a kinase upstream of NF-κB, MSK1, but not its upstream MAPK activators. This mechanism seems in contrast with a recently described mechanism in macrophages, explaining anti-inflammatory effects of single GCs not solely via gene suppression but through cooperative actions with p38 MAPK- and MSK1-dependent pathways, culminating in the upregulation and activation of another kinase, Sphingosine kinase 1 (SphK1) (41). However, these mechanisms do not necessarily exclude each other and are likely complementary. Indeed, it is not unreasonable to infer that different GC-assisted mechanisms may come in at different phases of the inflammatory response, or that in different cell types GCs may preferentially impact at different levels to establish a net anti-inflammatory effect. From our data, both GCs and PPARα agonist alone are able to partially drive MSK1 kinase from the nucleus, confirming earlier findings for GCs (23). At any rate, the finding that the subcellular distribution of MSK1 upon DEX/GW/TNF is similar to DEX/TNF implies that extrusion by itself is probably not a main mechanism explaining the additive gene repression. Rather, inhibition of MSK1 activation, which will hamper MSK1 activity, and interference at the level of NF-κB further downstream seem sufficient mechanisms to achieve additive cytokine gene repression (model in Figure 9). Taken together, it is clear that anti-inflammatory pathways that jointly tackle pathways leading to NF-κB activity will have an added advantage, as also found before in a study combining GCs with MSK1 inhibitors (42). Of interest from a clinical perspective, increased levels of activated MSK1 were detected in circulating blood CD14+ cells from patients with steroid-resistant asthma as compared to samples from steroid-sensitive asthma patients, linking a potential involvement of MSK1 in the regulation of cellular steroid responses (43). In a recent study in support of combination strategies, the team of Goleva showed benefit upon combining GCs with vitamin D, by demonstrating anti-inflammatory and GC-enhancing effects in monocytes of patients not only in steroid-sensitive asthma but also to some extent in steroid-resistant asthma (44).

We found that combined DEX/GW was able to reduce not only GRα and PPARα protein levels but also p65, in absence and presence of TNF. Regardless, inflammatory gene repression by combined GRα-PPARα agonists (studied here at the human IL8 promoter) was found to still involve maintaining the p65 subunit of NF-κB as well as GRα and PPARα at the chromatin (model in Figure 9). This finding apparently contrasts a study in macrophages showing GR activation, on its own, results in genome-wide blockade of NF-κB interaction with chromatin, as a late GC-induced event when inflammatory responses are allowed to fully mount (45). Again, this is not necessarily in conflict, as our study rather brings forward mechanisms likely to occur when GCs are ahead of a full-blown inflammatory response. In support of our data, in another recent study on mouse macrophages GR was rather shown to suppress pro-inflammatory gene expression by targeting distinct temporal events and components of transcriptional machinery in a gene-dependent manner, yet, the mechanism consistently involved a rapid GR tethering to p65 at NF-κB-binding sites (46). Our findings, adding PPARα to the equation, make it tempting to suggest a tripartite physical interaction mechanism may be possible. In line herewith, we retrieve all activated proteins (p65, GR, and PPARα) in the nuclear compartment, when performing pairwise indirect immunofluorescence of endogenous proteins in A549. Support for a physical interaction between p65, GRα and PPARα, at least *in vitro*, was found through non-competitive associations in GST-pull down analyses. Our data only shed light on a little piece of the anti-inflammatory mechanism following combined action of GRα and PPARα. Combined GRα/PPARα treatment reduces MSK1 kinase activation and appears to change the balance between nuclear vs. cytoplasmic MSK1, perhaps by preventing the accessibility of the kinase to the NF-κB target. Although these events clearly do not affect promoter recruitment of p65 or of pol II, at least not for IL8, a change in the activity status of NF-κB may well change coregulator associations, leading to a negative impact on gene expression. The *in vitro* interaction data, involving bacterial proteins and *in vitro* translated protein, suggest GR/PPAR/p65 complex formation, at least *in vitro*, might not be dependent on phosphorylation events, which is supported by the finding from the cell data that activated p65 remains efficiently recruited in presence of co-activated GRα/PPARα. It remains to be studied however, how frequent GRα and PPARα may co-localize in the cell models we have presented here, when subject to an inflammatory stimulus. In addition, direct proof of *in cellulo* complex formation at relevant promoter regions awaits firm evidence, for instance upon using re-ChIP experiments. Also the nature of the predominant binding sites remains to be investigated (half-site or palindromic GRE vs. PPRE vs. NF-κB response elements). In line with a previously recognized role for GRIP1 acting as a corepressor contributing to the suppressive action of GR (47–49), it is of current also unclear which cofactors may differentially associate with the GRα/PPARα co-suppressed inflammatory promoters as compared to either stimulus alone. On the physiological side, follow-up studies will have to demonstrate a predicted improved therapeutic benefit may take place, when co-administering GCs and PPARα agonists in an animal model of chronic inflammation (e.g., multiple sclerosis, arthritis, or asthma). Such study will allow simultaneous evaluation of the anti-inflammatory activity in relevant inflammatory target cells (depending on the animal model) with a metabolic impact addressing responses of the liver, regulating glucose and fat metabolism, when allowed to communicate with the other endocrine tissue within a complex organism under chronic inflammatory pressure.

## Data Availability

RNA-seq data have been submitted to the ArrayExpress tool (https://www.ebi.ac.uk/fg/annotare/) under the accession numbers E-MTAB-7296.

## Ethics Statement

Experiments were approved by the animal ethics committee of the Faculty of Medicine and Health Sciences at the University of Ghent (code dossiers 14/84 and 17/13).

## Author Contributions

NB and VM conducted experiments, analyzed data, and wrote parts of the manuscript. DR conducted experiments and contributed to the data analysis. IB revised the manuscript and advised on some experiments. LDC and JTh conducted experiments. JTa, BS, and CL contributed to the discussion section. KDB designed and supervised the research, conducted experiments, analyzed data, and wrote parts of the manuscript.

### Conflict of Interest Statement

The authors declare that the research was conducted in the absence of any commercial or financial relationships that could be construed as a potential conflict of interest.

## Supplementary Material

Figure S1 (Quantification Figure 2A). Western blot densitometric analysis. The P-IKK band (upper panel) and IκBα band (Lower panel) visualized via Western blot analysis in Figure 2A were subjected to band densitometric analysis using Image J. The amount of specific signal for P-IKK and IκBα was corrected to the respective tubulin loading control.

Figure S2 (Quantification Figures 3A,B). Western blot densitometric analysis. **(A)** The phospho-MAPK bands visualized via Western blot analysis in Figure 3A were subjected to band densitometric analysis (Image J). The amount of specific signal for phospho-MAPK was corrected to the respective corresponding non-phospho-MAPK signal. **(B)** The phospho-MSK1 bands visualized via Western blot analysis in Figure 3B were subjected to band densitometric analysis using Image J. The amount of specific signal for the phospho-MSK1 was corrected to the respective non-phospho-MSK1.

Figure S3 Ligand-activated GRα and p65 are both localized in the nucleus in TNF-stimulated cells. **(A)** A549 cells, starved for 48 h in DMEM devoid of serum, were pretreated with solvent, DEX (1 μM), GW (0.5 μM) or various combinations for 1h, before TNF (2000 IU/ml) was added, where indicated, for 30 min. Localization of p65 (green) and GRα (red) was assessed by confocal analysis. DAPI staining indicates the nuclei of the cells (blue). Immunofluorescence of representative cell fields is shown (*n* = 1). **(B)** Per induction, minimally three random fields of minimally 5 cells/field were scored. Scored cells are categorized into three groups according to the subcellular distribution of p65 (green) and GRα (red), i.e., C, mainly cytoplasmic; N, mainly nuclear; N/C, equally distributed (nuclear/cytoplasmic).

Figure S4 Ligand-activated PPARα and p65 are both localized in the nucleus in TNF-stimulated cells. **(A)** A549 cells, starved for 48 h in DMEM devoid of serum, were pretreated with solvent, DEX (1 μM), GW (0.5 μM) or various combinations for 1h, before TNF (2000 IU/ml) was added, where indicated, for 30 min. Localization of PPARα (green) and p65 (red) was assessed by confocal analysis. DAPI staining indicates the nuclei of the cells (blue). Immunofluorescence of representative cell fields is shown (*n* = 1). **(B)** Per induction, minimally three random fields of minimally 5 cells/field were scored. Absolute amounts of cells that were scored per induction were between 30 and 65 cells and categorized into three groups according to the subcellular distribution of PPARα (green) and p65 (red), i.e., C, mainly cytoplasmic; N, mainly nuclear; N/C, equally distributed (nuclear/cytoplasmic).

Figure S5 (Quantification Figures 6A–C). GST pull down analysis. **(A)** [^35^S]-methionine labeled GRα pull down and **(B,C)** [^35^S]-methionine labeled p65 and [^35^S]-methionine labeled GRα pull down visualized via autoradiography in Figure 5 were quantified using ImageJ analysis. Signals were normalized against respective inputs.

Figure S6 Combined PPARα/GRα activation diminishes p65 levels as well as nuclear receptor levels following 6h inductions, in absence and presence of TNF. **(A)** A549 cells, starved for 48 h in DMEM devoid of serum, were pretreated with solvent, DEX (1 μM), GW (0.5 μM) or various combinations for 1h, before TNF (2000 IU/ml) was added, where indicated, for a total induction time of 6h. Cell lysates were subjected to western blotting to detect GRα, PPARα or p65. Detection of β-actin served as a loading control. *n* = 1. **(B)** The bands visualized via Western blot analysis were subjected to band densitometric analysis using Image J. The amount of specific signal for was corrected to the respective actin loading control.

Figure S7 Combined PPARα/GRα activation already diminishes p65 levels as well as nuclear receptor levels following 1.5 h inductions, in absence and presence of TNF. **(A)** A549 cells, starved for 48 h in DMEM devoid of serum, were pretreated with solvent, DEX (1 μM), GW (0.5 μM) or various combinations for 1 h, before TNF (2000 IU/ml) was added, where indicated, for a total induction time of 1.5 h. Cell lysates were subjected to western blotting to detect GRα, PPARα or p65. Detection of β-actin served as a loading control. *n* = 1. **(B)** The bands visualized via Western blot analysis were subjected to band densitometric analysis using Image J. The amount of specific signal was corrected to the respective actin loading control.

Figure S8 Co-activation of GRα and PPARα enhances the lipid metabolism gene *Angptl4*. Gene counts for *Angptl4* upon DEX (1 μM), GW (0.5 μM) and LPS (100ng/ml) treatment of primary hepatocytes, from the experiment described in Figure 7. *n* = 1. Bars represent mean+SEM. NI, non-induced.

Table S1 List of qPCR primers.
