# Supplementary figures and images for: Mechanisms Underlying the Functional Cooperation Between PPARα and GRα to Attenuate Inflammatory Responses
Source: Front Immunol. 2019 Aug 9;10:1769. doi: 10.3389/fimmu.2019.01769 (PMC6695567; doi:10.3389/fimmu.2019.01769)

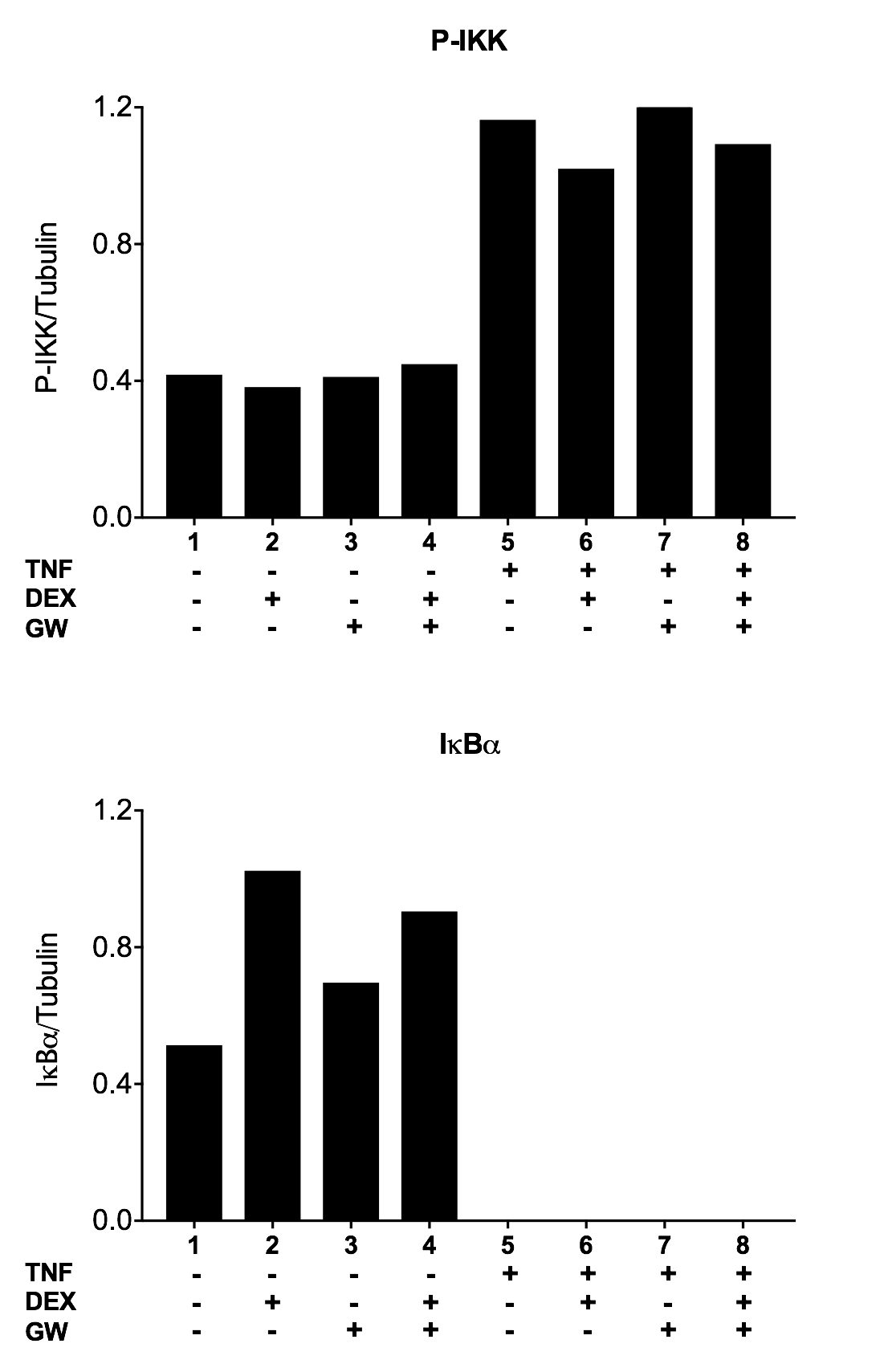

Supplement: Figure S1 — (Quantification Figure 2A). Western blot densitometric analysis. The P-IKK band (upper panel) and IκBα band (Lower panel) visualized via Western blot analysis in Figure 2A were subjected to band densitometric analysis using Image J. The amount of specific signal for P-IKK and IκBα was corrected to the respective tubulin loading control. [file Image_1.TIFF]

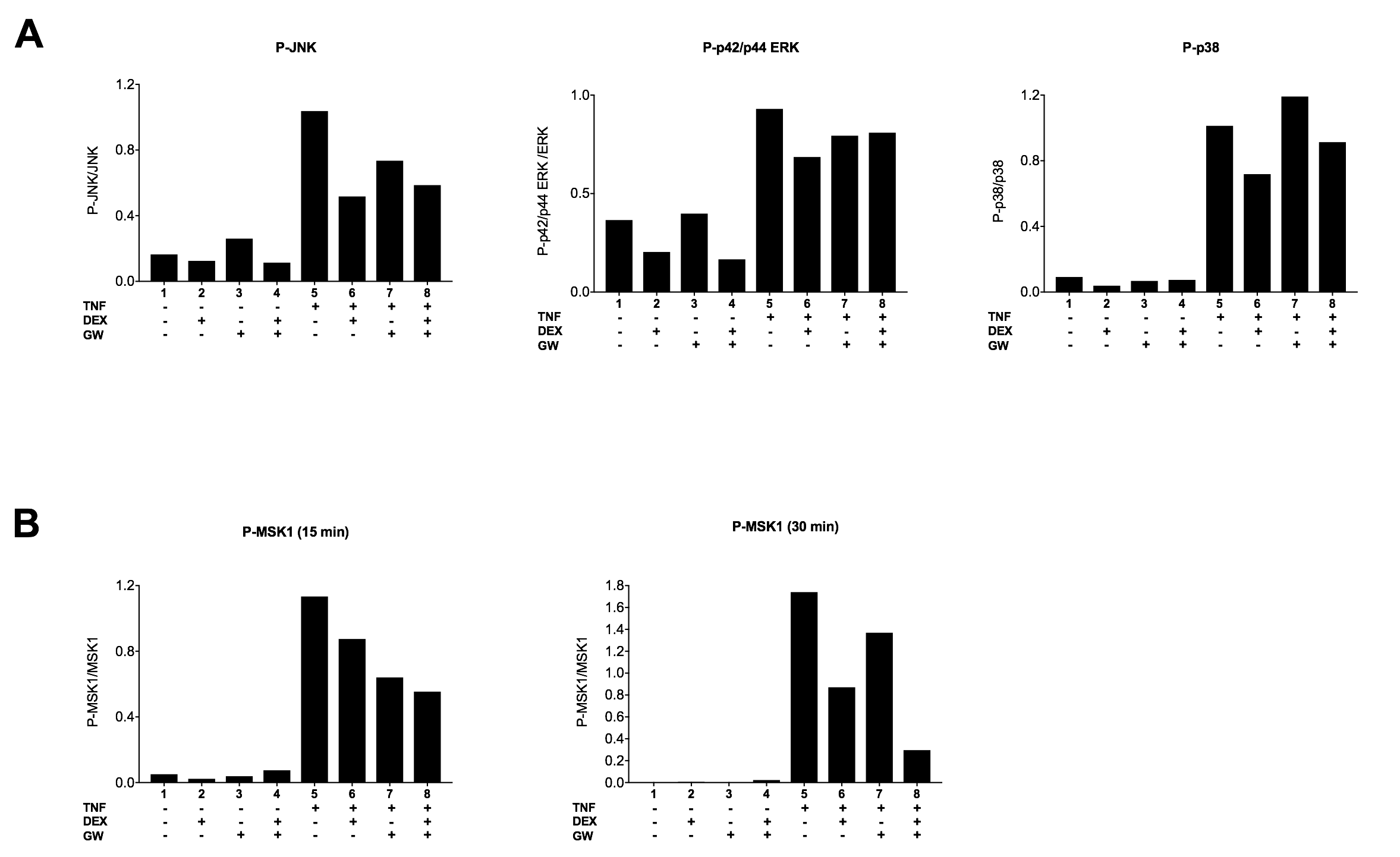

Supplement: Figure S2 — (Quantification Figures 3A,B). Western blot densitometric analysis. (A) The phospho-MAPK bands visualized via Western blot analysis in Figure 3A were subjected to band densitometric analysis (Image J). The amount of specific signal for phospho-MAPK was corrected to the respective corresponding non-phospho-MAPK signal. (B) The phospho-MSK1 bands visualized via Western blot analysis in Figure 3B were subjected to band densitometric analysis using Image J. The amount of specific signal for the phospho-MSK1 was corrected to the respective non-phospho-MSK1. [file Image_2.TIFF]

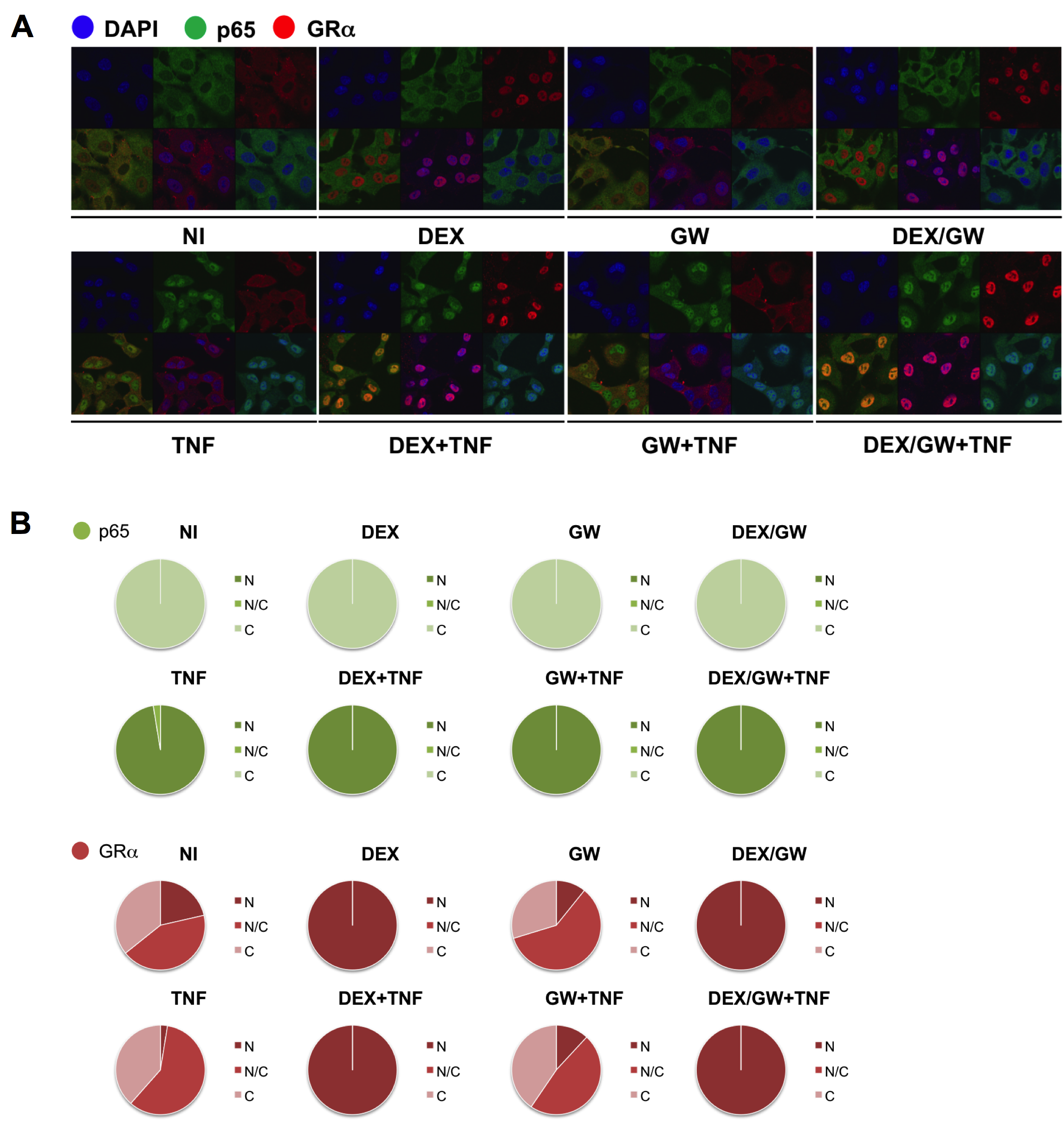

Supplement: Figure S3 — Ligand-activated GRα and p65 are both localized in the nucleus in TNF-stimulated cells. (A) A549 cells, starved for 48 h in DMEM devoid of serum, were pretreated with solvent, DEX (1 μM), GW (0.5 μM) or various combinations for 1h, before TNF (2000 IU/ml) was added, where indicated, for 30 min. Localization of p65 (green) and GRα (red) was assessed by confocal analysis. DAPI staining indicates the nuclei of the cells (blue). Immunofluorescence of representative cell fields is shown (n = 1). (B) Per induction, minimally three random fields of minimally 5 cells/field were scored. Scored cells are categorized into three groups according to the subcellular distribution of p65 (green) and GRα (red), i.e., C, mainly cytoplasmic; N, mainly nuclear; N/C, equally distributed (nuclear/cytoplasmic). [file Image_3.tiff]

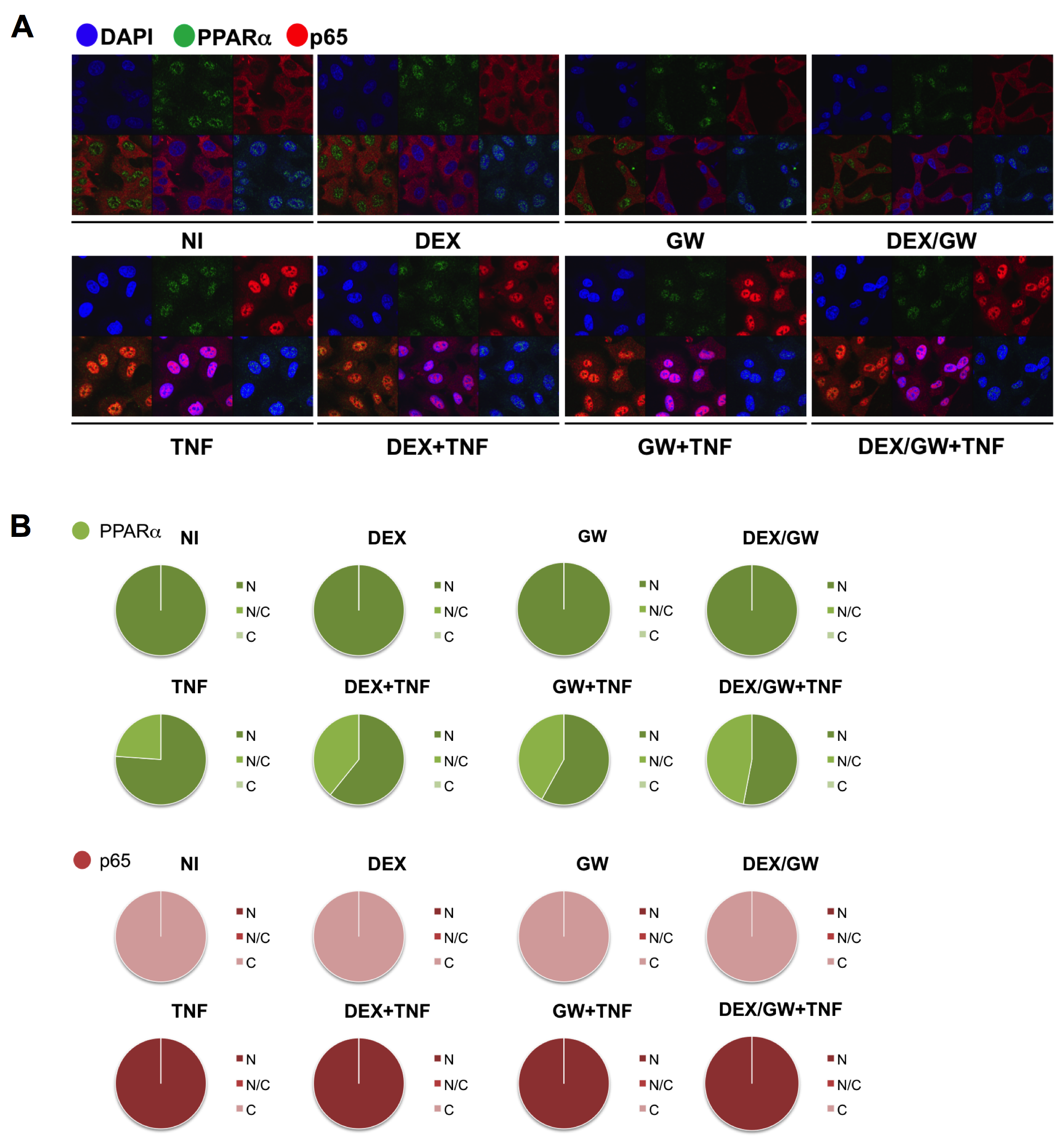

Supplement: Figure S4 — Ligand-activated PPARα and p65 are both localized in the nucleus in TNF-stimulated cells. (A) A549 cells, starved for 48 h in DMEM devoid of serum, were pretreated with solvent, DEX (1 μM), GW (0.5 μM) or various combinations for 1h, before TNF (2000 IU/ml) was added, where indicated, for 30 min. Localization of PPARα (green) and p65 (red) was assessed by confocal analysis. DAPI staining indicates the nuclei of the cells (blue). Immunofluorescence of representative cell fields is shown (n = 1). (B) Per induction, minimally three random fields of minimally 5 cells/field were scored. Absolute amounts of cells that were scored per induction were between 30 and 65 cells and categorized into three groups according to the subcellular distribution of PPARα (green) and p65 (red), i.e., C, mainly cytoplasmic; N, mainly nuclear; N/C, equally distributed (nuclear/cytoplasmic). [file Image_4.tiff]

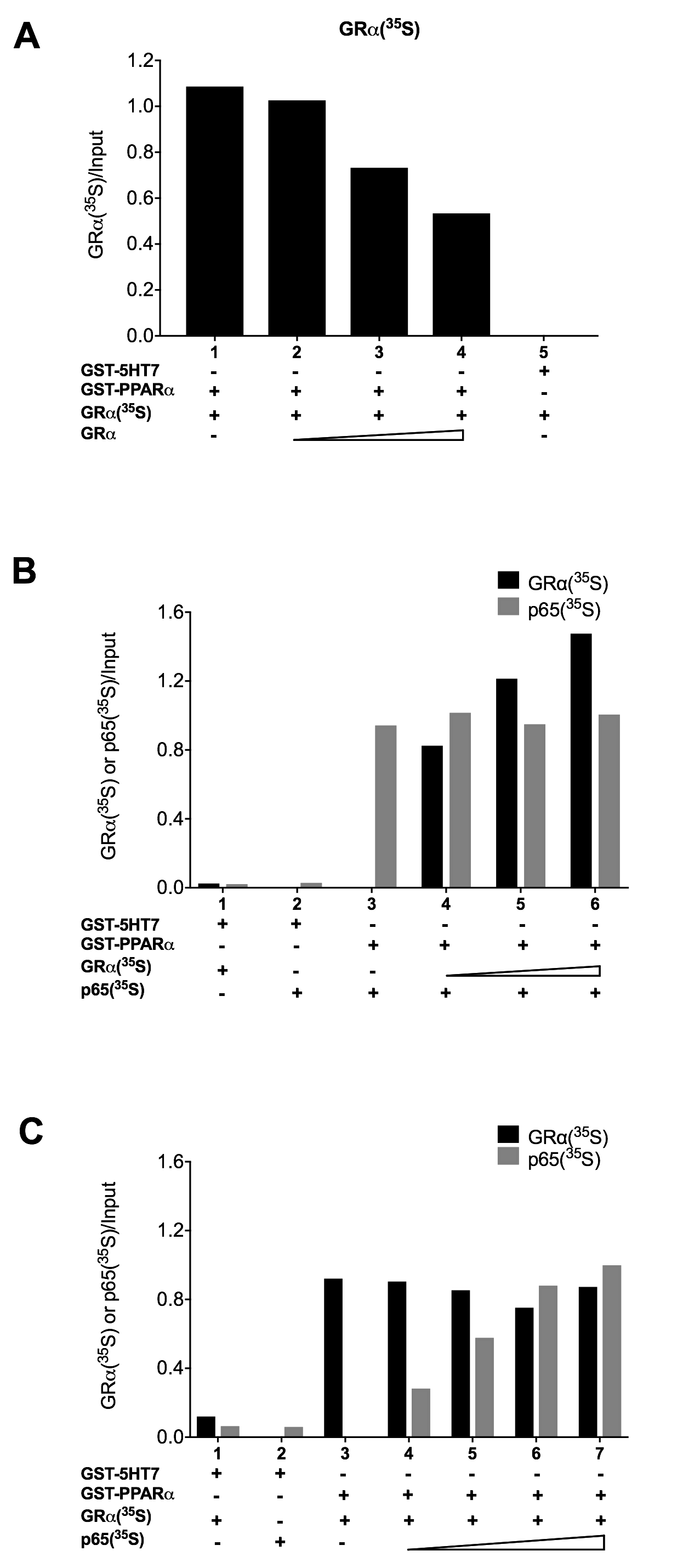

Supplement: Figure S5 — (Quantification Figures 6A–C). GST pull down analysis. (A) [35S]-methionine labeled GRα pull down and (B,C) [35S]-methionine labeled p65 and [35S]-methionine labeled GRα pull down visualized via autoradiography in Figure 5 were quantified using ImageJ analysis. Signals were normalized against respective inputs. [file Image_5.TIFF]

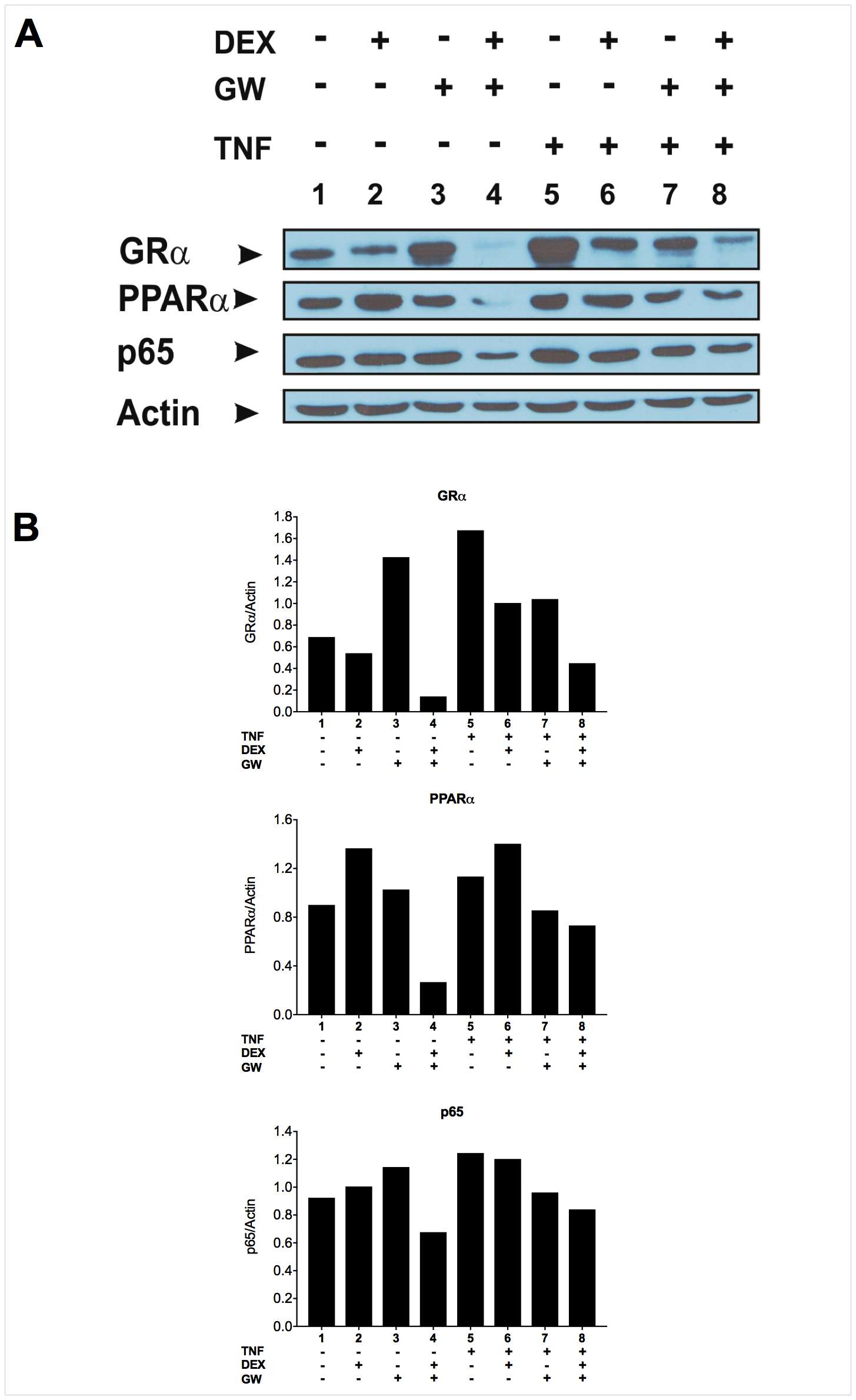

Supplement: Figure S6 — Combined PPARα/GRα activation diminishes p65 levels as well as nuclear receptor levels following 6h inductions, in absence and presence of TNF. (A) A549 cells, starved for 48 h in DMEM devoid of serum, were pretreated with solvent, DEX (1 μM), GW (0.5 μM) or various combinations for 1h, before TNF (2000 IU/ml) was added, where indicated, for a total induction time of 6h. Cell lysates were subjected to western blotting to detect GRα, PPARα or p65. Detection of β-actin served as a loading control. n = 1. (B) The bands visualized via Western blot analysis were subjected to band densitometric analysis using Image J. The amount of specific signal for was corrected to the respective actin loading control. [file Image_6.TIFF]

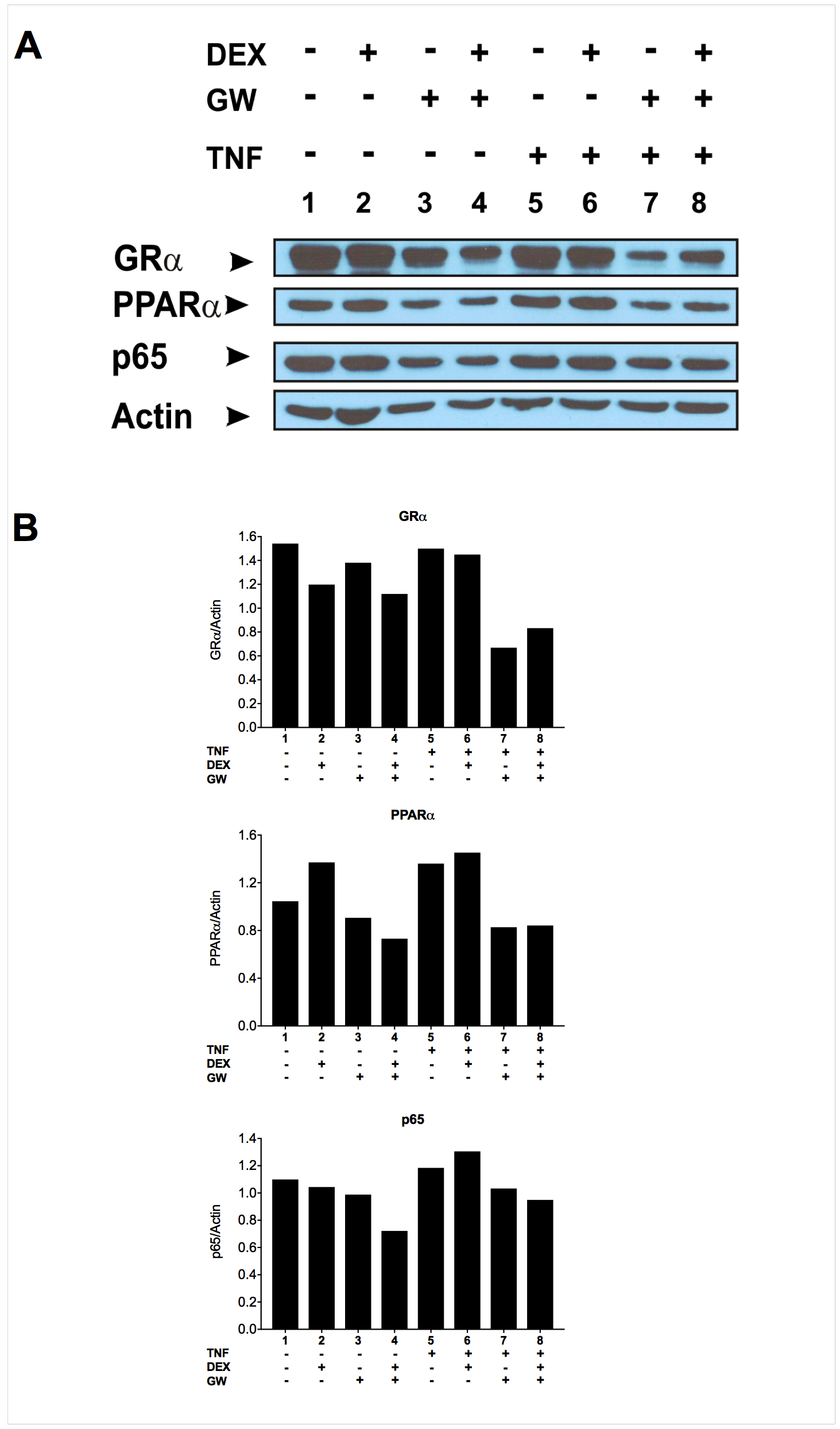

Supplement: Figure S7 — Combined PPARα/GRα activation already diminishes p65 levels as well as nuclear receptor levels following 1.5 h inductions, in absence and presence of TNF. (A) A549 cells, starved for 48 h in DMEM devoid of serum, were pretreated with solvent, DEX (1 μM), GW (0.5 μM) or various combinations for 1 h, before TNF (2000 IU/ml) was added, where indicated, for a total induction time of 1.5 h. Cell lysates were subjected to western blotting to detect GRα, PPARα or p65. Detection of β-actin served as a loading control. n = 1. (B) The bands visualized via Western blot analysis were subjected to band densitometric analysis using Image J. The amount of specific signal was corrected to the respective actin loading control. [file Image_7.TIFF]

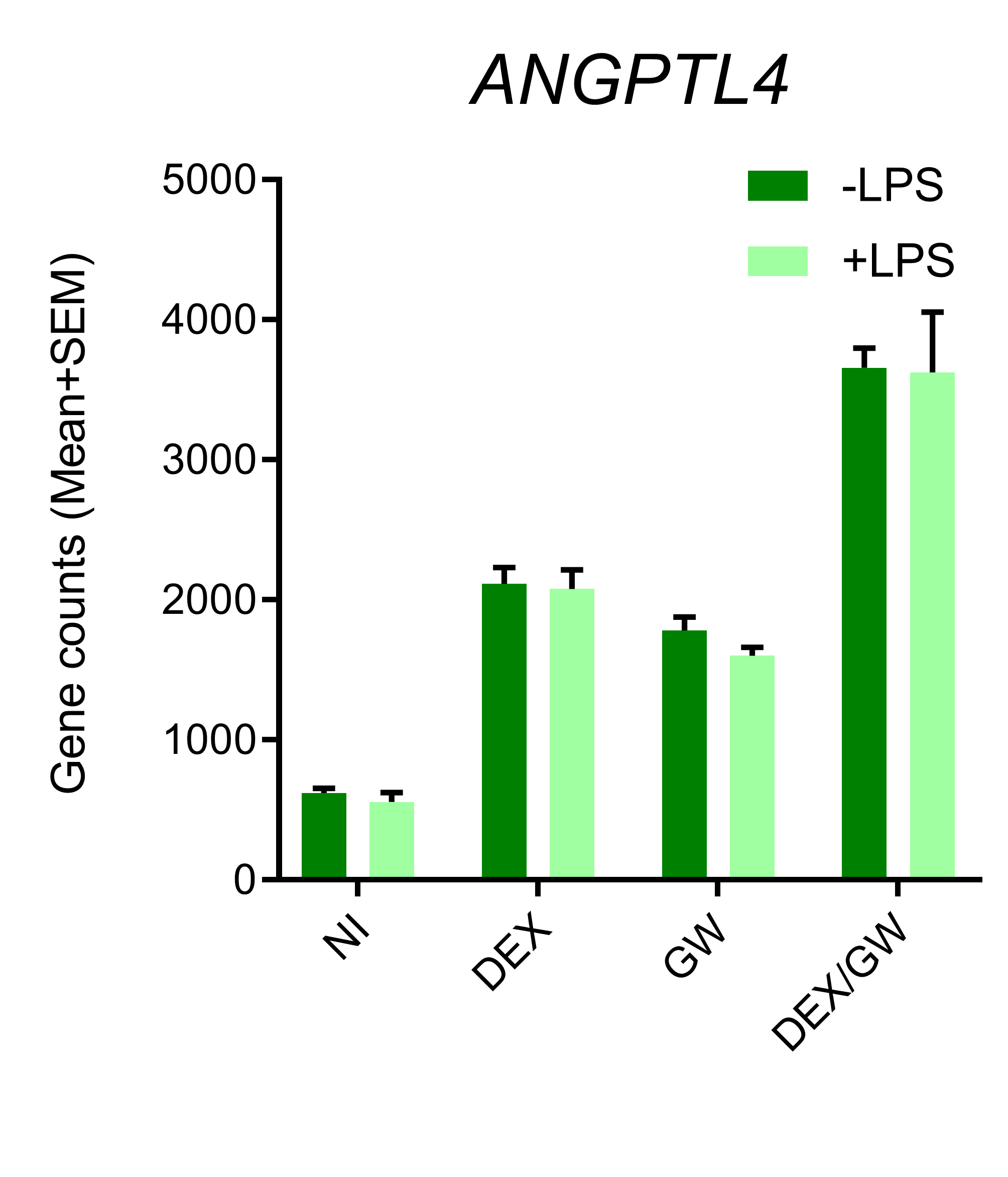

Supplement: Figure S8 — Co-activation of GRα and PPARα enhances the lipid metabolism gene Angptl4. Gene counts for Angptl4 upon DEX (1 μM), GW (0.5 μM) and LPS (100ng/ml) treatment of primary hepatocytes, from the experiment described in Figure 7. n = 1. Bars represent mean+SEM. NI, non-induced. [file Image_8.TIF]
