# Supplementary material for: Mechanisms Underlying the Functional Cooperation Between PPARα and GRα to Attenuate Inflammatory Responses
Source: Front Immunol. 2019 Aug 9;10:1769. doi: 10.3389/fimmu.2019.01769 (PMC6695567; doi:10.3389/fimmu.2019.01769)
Supplement: Table S1 — List of qPCR primers. [file Table_1.docx]

**Table S1. List of** **qPCR primers**.

| Target | Forward | Reverse |
| --- | --- | --- |
| *Ppia/Cyclophilin* | ATGGTGATCTTCTTGCTGGTCCTTGC | GCATACGGGTCCTGGCATCTTGTCC |
| *Gapdh* | AACTTTGGCATTGTGGAAGG | ACACATTGGGGGTAGGAACA |
| *Angptl4* | GGAAAGAGGCTTCCCAAGATG | CGTTGGGAGTCAAGCCAATG |
| *Ccl2* | TTAAAAACCTGGATCGGAACCAA | GCATTAGCTTCAGATTTACGGGT |
| *Nfkb2* | TGACTGTGGAGCTGAAGTGG | AAGGAGGCGAGTAAGAGTTGG |
| *Icam1* | TGCCTCTGAAGCTCGGATATAC | TCTGTCGAACTCCTCAGTCAC |
| *Tlr2* | GCAAACGCTGTTCTGCTCAG | AGGCGTCTCCCTCTATTGTATT |
| *Ikbke* | AGGATAAGTGCTGGGGCTTT | CCCTCGAAGAGGTACTCCTG |
| *Mapk3* | TCCAAGGGCTACACCAAATC | GTTTTCGAGGGCAGAGACTG |
| *IL8* | GCTGTCTTGGCAGCCTTCCTGA | ACAATAATTTGTGTGTTGGCGC |
